# Heterostructures Based on Cobalt Phthalocyanine Films Decorated with Gold Nanoparticles for the Detection of Low Concentrations of Ammonia and Nitric Oxide

**DOI:** 10.3390/bios12070476

**Published:** 2022-06-30

**Authors:** Svetlana I. Dorovskikh, Darya D. Klyamer, Evgeny A. Maksimovskiy, Victoria V. Volchek, Sergey M. Zharkov, Natalia B. Morozova, Tamara V. Basova

**Affiliations:** 1 Nikolaev Institute of Inorganic Chemistry SB RAS, 3 Lavrentiev Pr., 630090 Novosibirsk, Russia; reter16@yandex.ru (S.I.D.); klyamer@niic.nsc.ru (D.D.K.); eugene@niic.nsc.ru (E.A.M.); volchek@niic.nsc.ru (V.V.V.); mor@niic.nsc.ru (N.B.M.); 2 Kirensky Institute of Physics, Federal Research Center KSC SB RAS, 660036 Krasnoyarsk, Russia; zharkov@iph.krasn.ru; 3 Laboratory of Electron Microscopy, Siberian Federal University, 660041 Krasnoyarsk, Russia

**Keywords:** gold nanoparticles, cobalt phthalocyanine, gas-phase deposition, chemiresistive sensors, nitric oxide, ammonia

## Abstract

This work is aimed at the development of new heterostructures based on cobalt phthalocyanines (CoPc) and gold nanoparticles (AuNPs), and the evaluation of the prospects of their use to determine low concentrations of ammonia and nitric oxide. For this purpose, CoPc films were decorated with AuNPs by gas-phase methods (MOCVD and PVD) and drop-casting (DC), and their chemiresistive sensor response to low concentrations of NO (10–50 ppb) and NH_3_ (1–10 ppm) was investigated. A comparative analysis of the characteristics of heterostructures depending on the preparation methods was carried out. The composition, structure, and morphology of the resulting hybrid films were studied by X-ray photoelectron spectroscopy (XPS) and inductively coupled plasma atomic emission (ICP-AES) spectroscopy, as well as electron microscopy methods to discuss the effect of these parameters on the sensor response of hybrid films to ammonia and nitric oxide. It was shown that regardless of the fabrication method, the response of Au/CoPc heterostructures to NH_3_ and NO gases increased with an increase in the concentration of gold. The sensor response of Au/CoPc heterostructures to NH_3_ increased 2–3.3 times compared to CoPc film, whereas in the case of NO it increased up to 16 times. The detection limits of the Au/CoPc heterostructure with a gold content of ca. 2.1 µg/cm^2^ for NH_3_ and NO were 0.1 ppm and 4 ppb, respectively. It was shown that Au/CoPc heterostructures can be used for the detection of NH_3_ in a gas mixture simulating exhaled air (N_2_—74%, O_2_—16%, H_2_O—6%, CO_2_—4%).

## 1. Introduction

Gas sensors have a wide range of applications in various fields, including the chemical industry, food and cosmetics production, and agriculture and environment monitoring. Another important area of application for gas sensors is non-invasive medical diagnostics. The exhaled breath contains a large number of volatile organic compounds (VOCs), sulfur-containing compounds, halogen-containing compounds, nitrogen-containing compounds, etc. [1]. Among the gases considered as biomarkers for different medical conditions, the most important are ammonia, nitric oxide, hydrogen sulfide, acetone, ethane, and aldehydes. Recently, interest in the study of exhaled breath as a non-invasive method of diagnosing diabetes mellitus, renal failure, bronchopulmonary and cardiovascular disease, and other ailments has increased in the world [2,3,4]. These platforms are based on the analysis of the exhaled air of patients and the determination of biomarkers indicating the course of disease in these samples. In particular, an increase in the concentration of ammonia in exhaled air indicates the course of acute or chronic renal failure [5], and the accumulation of NO indicates the course of inflammatory processes in the respiratory tract [6,7,8,9]. In the breath of healthy people, the concentration of ammonia is in the range of 0.4–2 ppm [10]. A higher level of ammonia indicates kidney dysfunction. An increased concentration of ammonia in breath is also detected in people suffering from pulmonary arterial hypertension. The level of nitric oxide in the breath of healthy people is below 25 ppb, and elevated concentrations are found in the exhaled air of patients suffering from lung diseases [10].

Some important milestones have been reached in the field of human exhaled air analytics; however, only a few breath tests are used in clinical practice [11,12,13]. Currently, electrochemical and luminescence sensors, and gas chromatography–mass spectrometry techniques are used for exhaled breath analysis [14,15,16]. In addition, Pan et al. described an application of the analyzer based on electrospray ionization mass spectrometry for the detection of NO in exhaled breath [15]. However, these techniques require bulky and expensive equipment and complex sample preparation. Generally, semiconductor sensors are cheaper, more portable, and faster. The combination of some cross-reactive chemiresistive sensor layers in arrays by applying pattern recognition and classification algorithms is a well-known, promising approach for the detection of gas mixtures [17].

Recently, it was shown that nanomaterials (CNTs, metal oxides, and noble metals) can be used for clinical diagnostics by breath analysis because of their chemiresistive sensor response, porous structure, large surface-area-to-volume ratio, and comparatively simple preparation techniques [17,18]. An overview of the potential application of various carbon nanotube-based hybrid materials with metal oxides, nanoparticles, polymers, or organometallic compounds for biologically relevant breath markers is given in the review of J. Ellis et al. [19]. For example, the combination of CNTs with conducting polymers such as polypyrrole or polyaniline led to the improvement of the sensitivity and selectivity of the nanocomposites to ammonia in the presence of H_2_S, NO_2_, acetone, ethanol, and isoprene.

A chemiresistive electronic nose based on an array of metal oxide thin films, including WO_3_, SnO_2_, In_2_O_3_, and Bi_2_O_3_, was also suggested in some papers [20,21,22]. For example, Moon et al. [23] used chemiresistive porous WO_3_ thin films and achieved the selective and sensitive detection of the asthma biomarker NO in the presence of ethanol, acetone, NH_3_ and CO at 150–250 °C, with an LOD of 88 ppb, which is far lower than that of exhaled NO.

Catalytic nanoparticles (e.g., Pt, Pd, Au, and Ag) are widely used for the functionalization of the metal oxide and carbon nanomaterials, leading to the enhancement of their sensitivity and selectivity due to the spillover effect [24]. There are examples of the use of Pt-functionalized SnO_2_ fibers and Au-functionalized In_2_O_3_ layers for the detection of volatile organic compounds as biomarkers of diabetes and lung cancer [25,26]. However, it is worth mentioning that the reversibility of the metal-oxide-based active layer can be reached only at high temperatures.

Although human breath analyzers based on the above-mentioned nanomaterials have never been commercialized, the analysis of the literature shows that the area of research is progressing rapidly. The main bottleneck here is the creation of sensors with high sensitivity (detection limits down to ppb and lower) and selectivity because of interferences between the components of exhaled breath, a highly humid environment. Therefore, the search for reliable materials with unique performance characteristics such as sensitivity and selectivity is a crucial task.

Metal phthalocyanines are among the most demanded sensor materials, which have attracted the attention of researchers because of their ability to change conductivity in the presence of various gases due to charge transfer reactions, as well as because of their high thermal and chemical stability compared to most organic materials [27,28,29]. Unlike most metal oxide sensors, the phthalocyanine-based active layers exhibit a chemiresistive sensor response at room temperature with a recovery time of no more than a few minutes [30]. These properties make the layers of metal phthalocyanines competitive with other materials, which are used in chemiresistive sensors for the detection of such gases as NH_3_, H_2_, H_2_S, and NO_x_; however, phthalocyanine films have not yet been studied as sensors of gaseous biomarkers in exhaled air or similar gas mixtures. 

Noble metal nanoparticles and nanostructures are known to be widely used to improve the performance of chemiresistive sensors for the detection of various gases such as hydrogen [31], methane [32], taggants in explosives (2-nitrotoluene and 2,3-dimethyl-2,3-dinitrobutane), and NO_2_ [33]. Recent reports highlighted the advantages of sensors based on heterostructures due to the synergistic effect of their components [34,35,36]. The modification of the surface of semiconductors with noble metal cores allows the generation of additional adsorption centers, which can increase sensitivity to detectable gases by changing the electrical conductivity of the semiconductor or chemical sensitization mechanisms [37,38,39]. 

The synergetic combination of the properties of phthalocyanines and metal nanoparticles is known to lead to an improvement in the performance of both electrochemical [40] and chemiresistive [41,42] sensors. Recent studies showed that bilayer structures consisting of Pd and H_2_Pc [43] or various MPc (CuPc [44], PdPc [45], and VOPc [42]) demonstrated an improved sensor response to hydrogen compared to that of pure phthalocyanine films. Tomeček et al. [33] demonstrated a metal/silver phthalocyanine (AgPc) chemiresistive sensor for the detection of taggants in explosives (2-nitrotoluene and 2,3-dimethyl-2,3-dinitrobutane) and NO_2_ as a product of their photodecomposition. AgPc layers deposited by evaporation in vacuum were covered with noble metals (Pd, Ag, Au) by magnetron sputtering. It was shown that Pd(1 nm)/AgPc sensors exhibited high sensitivity to photoactivated 2-nitrotoluene and excellent recovery. 

To date, Au-containing hybrid materials are already used as materials for electrochemical sensors detecting glucose and NO metabolites [46,47,48], while the possibility of using these heterostructures for the detection of N-containing gases has not yet been sufficiently studied. Wet chemical methods [49,50,51] are traditionally used to fabricate AuNPs on various surfaces. The latest reports have shown the advantages of using gas-phase methods for the deposition of metal layers and nanoparticles on the surface of organic semiconductors to create sensing layers with good sensor performance [34,52,53]. The methods of metal–organic chemical vapor deposition (MOCVD) and physical vapor deposition (PVD) for the preparation of hybrid materials open up new possibilities for the creation of sensors with the desired characteristics. 

The main goal of this research is to develop new heterostructures based on cobalt phthalocyanines (CoPc) and gold nanoparticles (AuNPs) and to evaluate the prospects of their use to determine low concentrations of ammonia and nitric oxide, which are biomarkers of disease in exhaled air. For this purpose, CoPc films were decorated with AuNPs by various methods, viz. MOCVD, PVD, and drop-casting (DC), and their chemiresistive sensor response to low concentrations of NO (10–50 ppb) and NH_3_ (1–10 ppm) was investigated. A comparative analysis of the characteristics of heterostructures depending on the preparation methods was carried out. The composition, structure, and morphology of the resulting hybrid films were studied by X-ray photoelectron spectroscopy (XPS) and inductively coupled plasma atomic emission (ICP-AES) spectroscopy, as well as electron microscopy methods to discuss the effect of these parameters on the sensor response of hybrid films to ammonia and nitric oxide. To investigate the feasibility of the detection of ammonia in exhaled air using the investigated heterostructures, their sensor response to the main interfering analytes, which can present in the exhaled air, and their sensor response to ammonia in a mixture of gases with a composition close to the exhaled air (N_2_—74%, O_2_—16%, H_2_O—6%, CO_2_—4%) were tested.

## 2. Materials and Methods

### 2.1. Materials

**Scheme 1:**
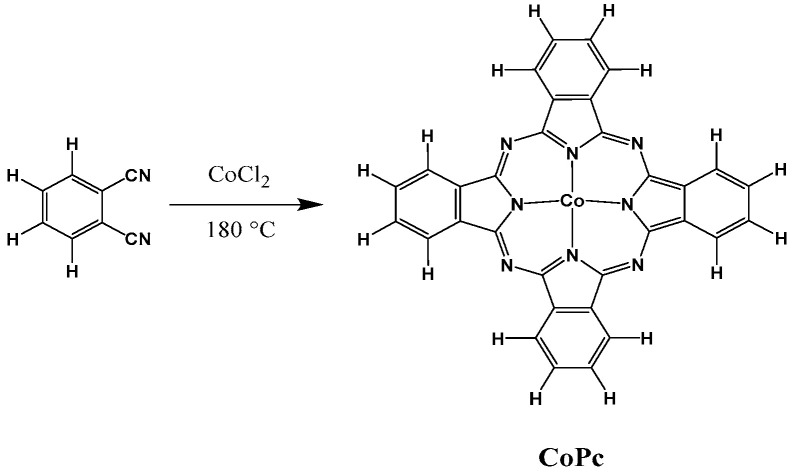
CoPc synthetic pathway.

The choice of CoPc was due to the fact that its exhibits a higher sensor response to nitrogen-containing analytes, such as NH_3_ and nitrogen oxides, compared to phthalocyanines of other metals. In addition, CoPc is highly stable and sublimates in vacuum without decomposition, and its films can be obtained by a PVD method. CoPc was synthesized by heating a mixture of phthalonitrile (CAS N 91-15-6, Sigma-Aldrich, Saint Louis, MO, USA) and cobalt chloride (CAS 7646-79-9, Sigma-Aldrich, Saint Louis, MO, USA) at 180 °C according to Scheme 1 [54]. 

CoPc: C_32_H_16_N_8_Co. Anal. Calc: C 67.3; H 2.8; N 19.6. Found: C 67.3; H 2.7; N 19.7. IR spectrum (KBr; ω, cm^−1^): 1609, 1591, 1522, 1468, 1425, 1333, 1288, 1165, 1121, 1088, 1001, 951, 912, 874, 779, 754, 571, 517, 434.

**Scheme 2:**
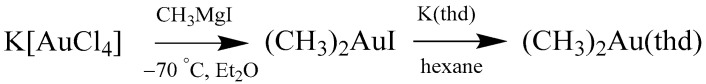
(CH_3_)_2_Au(thd) synthetic pathway.

[(CH_3_)_2_Au(thd)] (thd-2,2,6,6-tetramethyl-heptane-3,5-dionato(-)), which was chosen as a MOCVD precursor for the deposition of gold nanoparticles, was synthesized according to Scheme 2.

A total of 1 g (2.6 mmol) of K[AuCl_4_] was obtained by precipitation from a solution of H[AuCl_4_] hydrate (metal content ≥ 47.8%, Krastsvetmet, CAS 16903-35-8) after adding of 0.15 g (2.6 mmol) of KCl. The 0.35 g (1 mmol) of (CH_3_)_2_AuI was isolated with the yield of 40% at the interaction of 1 g (2.6 mmol) of K[AuCl_4_] and solution of 10 mmol of CH_3_MgI in 200 mL of cooled diethyl ether in the Schlenk apparatus. The 0.35 g (1 mmol) (CH_3_)_2_AuI dissolved in 10 mL of cooled hexane interacted with 0.22 g (1 mmol) of K(thd) obtained by neutralization of Hthd (97%, Dalchem, CAS 1118-71-4, Cheltenham, Australia) by KOH solution. The (CH_3_)_2_Au(thd) was purified by recrystallization from hexane; the yield was 65%: C_13_H_25_O_2_Au. Anal. Calc: C 38.1; H 6.1. Found: C 37.8; H 6.2. IR spectrum (KBr; ω, cm^−1^): 2980, 2951, 2900, 1568, 1525, 1433, 1208, 1165, 1008, 951, 922, 864, 754, 573, 527, the melting point was 74 °C [55].

Single-crystal Si(100) plates of 10 × 10 mm^2^ (Alga-SW, Novosibirsk, Russia), freshly cleaved single crystals of NaCl, glass plates of 20 × 20 mm^2^ (Deltalab, Barcelona, Spain), and glass slides with interdigitated Pt electrodes (IDE, Metrohm, Dropsens, Oviedo, Spain: the gaps dimension is 10 μm; the number of digits is 125 × 2 with a digit length equal to 6760 μm; cell constant is 0.0118 cm^−1^) were used as substrates. Pure commercial Ar, NH_3_, and NO gases were provided by the Company “Chistye Gasy”, Novosibirsk, Russia.

### 2.2. Preparation of Au/CoPc Heterostructures

**Figure 1:**
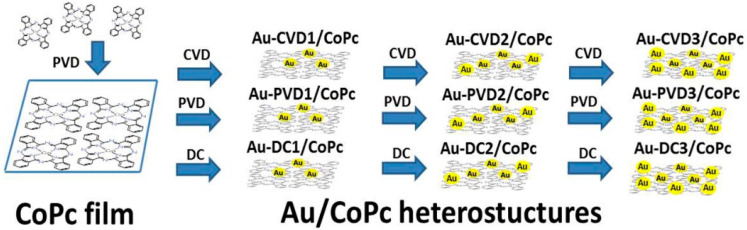
Scheme of the preparation of Au/CoPc heterostructures.

Au/CoPc heterostructures were obtained according to the general scheme shown in Figure 1.

First, CoPc films were prepared by the PVD method. The deposition conditions were as follows: the total pressure was 5 × 10^−5^ Torr, the evaporation temperature was 450 °C, the deposition time was 1 h, and the substrate temperature was 60 °C. It is known that when CoPc films are heated at temperatures above 200 °C, a phase transition from α-modification to β can occur, and the size of crystallites and their packing density can change, affecting the sensor properties. For this reason, before AuNPs deposition, CoPc films were annealed under the conditions of the MOCVD experiment (T = 280 °C, Ar/O_2_ = 2, 1 h) to obtain CoPc films with the same phase composition in order to exclude the effect of this parameter on the sensor properties.

Second, the surface of annealed CoPc films was decorated with AuNPs using three different methods, viz. MOCVD, PVD, or DC. AuNPs were deposited on annealed CoPc films by MOCVD using (CH_3_)_2_Au(thd) as a precursor in a reactor with cold walls, equipped with an Ar lamp. The evaporator temperature was 60 °C (the saturated vapor pressure of (CH_3_)_2_Au(thd) was given by the equation ln(p, atm) = 36.61–15,231/T(K) [55] corresponding to the precursor partial pressure 5 × 10^−2^ Torr). The deposition temperature was varied from 210 to 280 °C, and the optimal deposition temperature was 280 °C. Other experimental parameters were set as follows: the precursor weight was 3 mg, the ratio of the reactant gaseous mixture (Ar/O_2_) in the reaction zone was 2; the total reactor pressure was ~2 Torr; and the deposition time was 3 min. The deposition procedure was performed one, two, or three times sequentially, and the samples obtained in this way were designated as Au_CVD1/CoPc, Au_CVD2/CoPc, and Au_CVD3/CoPc, respectively (Figure 1).

AuNPs were deposited on annealed CoPc films by PVD from the load of metallic Au in vacuum (P = 10^−6^ Torr) using a UVM-71 installation. The load of gold was varied from 0.6 to 5 mg, and the load of 0.6 mg was chosen as optimal for obtaining samples. The evaporator temperature was 1532 °C (which corresponds to a partial pressure of Au 10^−2^ Torr), and the substrate temperature was 100 °C. The deposition procedures were carried out until complete evaporation of Au (0.6 mg). This procedure was repeated one, two, or three times sequentially, and the samples obtained in this way were designated as Au_PVD1/CoPc, Au_PVD2/CoPc and Au_PVD3/CoPc, respectively (Figure 1).

AuNPs were deposited on annealed CoPc films by DC of 5 × 10^−4^ M AuNPs solution in hexane, prepared according to the method proposed by Eah et al. [56]. In brief, 400 μL of 0.05 M of NaBH_4_ in an aqueous NaOH was added slowly to 100 μL of 0.05 M of HAuCl_4_·3H_2_O in aqueous HCl, which led to the formation of a red solution. The distilled water was added to obtain 5 × 10^−4^ M AuNPs solution. After that, 5 mL of acetone was added to 10 mL of 5 × 10^−4^ M AuNPs aqueous solution and the mixture was shaken manually for 2 min. Then, 0.1 g of dodecanethiol in 5 mL of hexane was added rapidly and the mixture was shaken manually for 2 min. The separation of the water and hexane layers was accompanied by discoloration of the lower aqueous phase and staining of the upper organic phase. The layer of hexane was collected. The new portion of 0.1 g of dodecanethiol in 5 mL of hexane was added to the water layer, and the above procedure was repeated 5 times. After combining the hexane layers, the total mixture was concentrated in a vacuum rotary evaporator. The addition of 5 mL of ethanol to the concentrated hexane solution resulted in the precipitation of dark amorphous solid of AuNPs. To remove dodecanethiol, the precipitate was dissolved again in hexane and re-precipitated by ethanol. Finally, the precipitate was dissolved in 10 mL of hexane, giving ~5×10^−4^ M AuNPs solution. One, two, or three drops (one drop corresponded to 1 µL) were dripped onto the surface of CoPc films and dried in an oven at 150 °C for 2 h to obtain Au_DC1/CoPc, Au_DC2/CoPc, and Au_DC3/CoPc samples, respectively (Figure 1).

### 2.3. Methods of Characterization of Heterostructures

The chemical state of gold in the heterostructures with AuNPs obtained by MOCVD and DC methods was investigated using X-ray photoelectron spectroscopy (XPS, SPECS Spectrometer, Berlin, Germany, PHOIBOS-150-MCD-9 analyzer, FOCUS-500 monochromator, Al Kα radiation, hv = 1486.74 eV, 200 W). The binding energy scale (E_b_) was calibrated using the positions of the peak energy levels of the Au 4f_7/2_ (E_b_ = 84.0 eV) and Cu 2p_3/2_ (E_b_ = 932.67 eV). The spectra were processed in the CASA program (Japan) using the Voigt function. The background was taken into account using the Shirley method. 

The content of gold in the samples Au_CVD1/CoPc, Au_CVD2/CoPc, Au_CVD3/CoPc, Au_PVD1/CoPc, Au_PVD2/CoPc, and Au_PVD3/CoPc was determined by inductively coupled plasma atomic emission spectroscopy (ICP-AES) using a high-resolution spectrometer iCAP 6500 Duo (Thermo Fisher Scientific, Waltham, MA, USA). HCl (ACS reagent, 37%), HNO_3_ (70%, purified by redistillation _99.999% trace metals basis), deionized water (purified with the Direct-Q3 system (Millipore, Burlington, MA, USA) > 18 MU/cm), Ar gas (99.999%) and Gold Standard for ICP TraceCERT^®^, 1000 mg/L Au in hydrochloric acid were used as reagents for the determination of gold content. The heterostructures (Au/CoPc) were washed off the surface of the samples of Au_CVD1/CoPc, Au_CVD2/CoPc, Au_CVD3/CoPc, Au_PVD1/CoPc, Au_PVD2/CoPc, and Au_PVD3/CoPc deposited on glass substrates using a minimal amount of concentrated HCl and HNO_3_ (3:1). The resulting solutions were injected into the plasma through a nebulizer of SeaSpray type using a peristaltic pump. The working parameters of the ICP-AES system are as follows: power supply—1150 W, nebulizer argon flow rate—0.70 L min^−1^, auxiliary—0.50 L min^−1^, and cooling—12 L min^−1^. The data acquisition and processing were carried out with iTEVA (Thermo Scientific, Philadelphia, PA, USA) software.

The elemental composition and surface morphology of heterostructures based on CoPc films and AuNPs obtained by CVD, PVD, and DC methods were investigated using scanning electron microscopes, JEOL–JSM 6700 F (connected with EDX-analizator EX-2300BU) and HITACHI UHR FE-SEM SU8200. The particle size distribution in the samples were determined using image analysis software (ImageJ, National Institutes of Health, 9000 Rockville Pike, Bethesda, Rockville, MA, USA). The composition and microstructure of Au_CVD1/CoPc and Au_DC1/CoPc samples deposited on a freshly cleaved NaCl crystal or a Cu grid were analyzed using transmission electron microscopy (TEM), electron diffraction, and energy-dispersive spectroscopy on a JEM-2100 transmission electron microscope (TEM, JEOL). The diffraction patterns were identified using the program DigitalMicrograph (Gatan, Pleasanton, CA, USA) and crystal structure database ICDD PDF 4+ (2020). 

### 2.4. Study of the Sensor Properties

The study of the sensor properties of active layers based on CoPc and its heterostructures with AuNPs was carried out by measuring their chemiresistive sensor response to NH_3_ and NO. CoPc films and heterostructures were deposited onto a glass substrate with interdigitated Pt electrodes. In this method, the measured value is the resistance of the film, which reversibly changes during the interaction of the layer with the gas to be determined and subsequent purging. The change in the resistance of hybrid film structures was measured using a Keithley 236 universal electrometer by applying a constant DC voltage (10 V). The sensor response (S_resp_) was estimated as S_resp_ = (R − R_o_)/R_o_, where R_o_ and R are the initial sensor resistances (base resistance) and the resistances in the presence of analyzed gas, respectively. The standard deviation of the response values on each line was calculated using the data for three different samples. 

**Figure 2:**
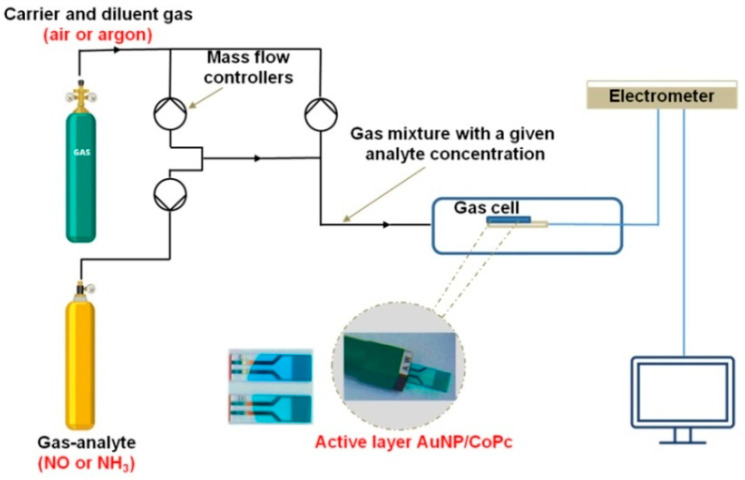
Scheme of the system for investigation of chemiresistive sensor response with a photograph of interdigitated electrodes.

The scheme of the system for investigation of the chemiresistive sensor response with a photo of the gas flow cell and the sensing layer deposited onto IDE is shown in Figure 2. 

Pure commercial NH_3_ and NO gases (Company “Chistye Gasy”, Novosibirsk, Russia) were used as analyte sources. Air was used as a carrier and diluent gas in the case of investigation of the sensor response to ammonia. Argon (Company “Chistye Gasy”, Novosibirks, Russia) was used as a carrier gas and diluent gas in the case of investigation of the sensor response to NO because of its instability in air. The analyzed gases were diluted with the corresponding diluent gases to the required concentration and passed through the cell at the constant flow rate of 300 mL∙min^ࢤ1^. The required gas flow was regulated using mass flow regulators. After the input of the analyzed gas, the cell was purged with an air (or argon) flow until the initial resistance was restored. To achieve complete reversibility of the sensor response to NO, measurements were carried out at the temperature of 40–50 °C. For a correct comparison of the sensor properties of the layers, studies of the sensor response to ammonia were also carried out when the gas-flow chamber was heated at the same temperature.

## 3. Results and Discussion

### 3.1. Characterization of Heterostructures with AuNPs Obtained by MOCVD

To obtain AuNPs on CoPc films, the experimental parameters of the MOCVD process were chosen in accordance with our previous results [57] on the deposition of AuNPs from (CH_3_)_2_Au(thd) onto Si plates. It was experimentally established that AuNPs were grown on the CoPc surface at a deposition temperature of at least 280 °C, while the subsequent increase in the temperature was accompanied by the degradation of the CoPc film. The time and the precursor load were chosen experimentally to ensure the formation of AuNPs in each of the Au_CVD1/CoPc, Au_CVD2/CoPc, and Au_CVD3/CoPc samples. 

The presence of gold in Au_CVD1/CoPc, Au_CVD2/CoPc, and Au_CVD3/CoPc was confirmed by ICP-AES data (Table 1). The concentration of gold in the sample Au_CVD1/CoPc was 0.34 ± 0.05 μg/cm^2^. Each subsequent deposition was accompanied by a uniform increase in the concentration of gold in the samples: 0.64 ± 0.08 and 1.1 ± 0.1 μg/cm^2^ for Au_CVD2/CoPc and Au_CVD3/CoPc, respectively. 

**Figure 3:**
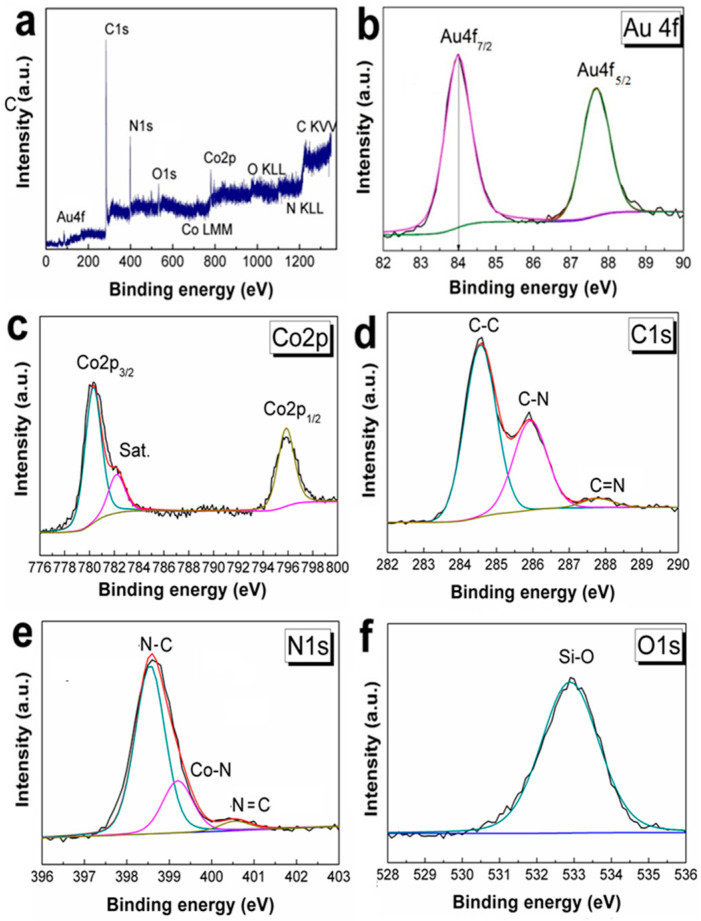
XPS spectra of Au_CVD3/CoPc (**a**), fitting of Au4f spectra (**b**), fitting of Co2p spectra (**c**), fitting of C1s spectra (**d**), fitting of N1s spectra (**e**), and fitting of O1s spectra (**f**).

Due to the low concentration of gold in Au_CVD1/CoPc, characteristic Au4f lines were absent in its XPS spectrum, but these lines were visualized in the XPS spectra of Au_CVD2/CoPc and Au_CVD3/CoPc. The XPS spectrum of Au_CVD3/CoPc is shown in Figure 3a. Along with Au4f lines, the intense Co2p, C1s, and N1s lines related to CoPc films, as well as low-intensity O1s and Si2p lines from the substrate, were observed in the XPS spectra of Au_CVD2/CoPc and Au_CVD3/CoPc samples. The positions of the main Au4f peaks in the XPS spectrum of Au_CVD3/CoPc indicated the presence of only metallic gold (Au4f_7/2_ 84.1 eV) in this sample (Figure 3b). This confirms the successful deposition of metallic gold on CoPc by the MOCVD method using [(CH_3_)_2_Au(thd)] as a precursor.

The Co2p can be resolved into two peaks, 2p3/2 and 2p1/2, at 780.3 (its satellite at 782.8 eV) and 796 eV, respectively (Figure 3c). The positions of these peaks are in good agreement with those for the annealed CoPc film described by Kumar et al. [58]. The XPS spectrum of C1s (Figure 3d) exhibited three peaks at about 284.6, 286, and 287.8 eV, assigned to C–C, C–N, and C=N, respectively. The N1s binding energy of the Au_CVD3/CoPc sample showed three types of nitrogen, and peaks at 398.5 and 400.5 eV were assigned to the nitrogen of the phthalocyanine ring [59], while the peak at 399.3 eV was ascribed to the coordination of cobalt and nitrogen (Figure 3e). Thus, there was no degradation of the CoPc film even after three consecutive MOCVD steps (Au_CVD3/CoPc sample). In the O1s spectrum (Figure 3f), oxygen was in a single state (532.9 eV), which could be attributed to Si-O [60].

**Figure 4:**
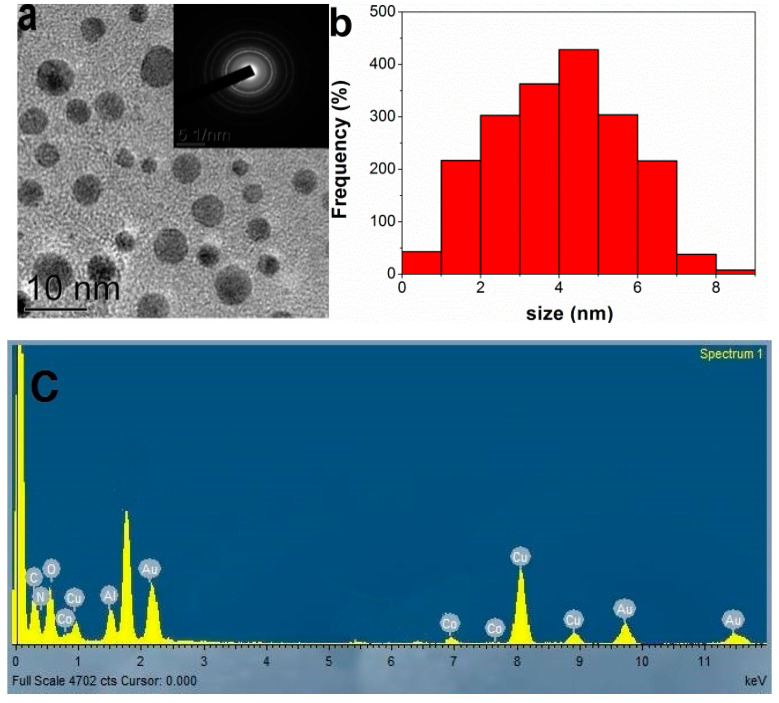
TEM images of Au_CVD1/CoPc (**a**) with size distribution (**b**) and EDX spectrum (**c**) (Cu lines refer to the TEM grid, and Al lines refer to the crucible for the sample).

The TEM method was used to study the composition and morphology of the heterostructures. Figure 4a shows a TEM image of the Au_CVD1/CoPc sample as an example. The diffraction pattern of Au_CVD1/CoPc contained sets of ring-type reflections from fcc-Au (JCPDS Card No. 04-0784) with polycrystalline structure and from the β-CoPc phase. The surface of the Au_CVD1/CoPc sample was formed by spherical AuNPs evenly distributed over the surface of the CoPc film. This sample was characterized by a unimodal size distribution of AuNPs with a predominant size of 5 nm (Figure 4b). According to the EDX spectrum, the gold content in Au_CVD1/CoPc was 2.8 at.% (Figure 4c). 

**Figure 5:**
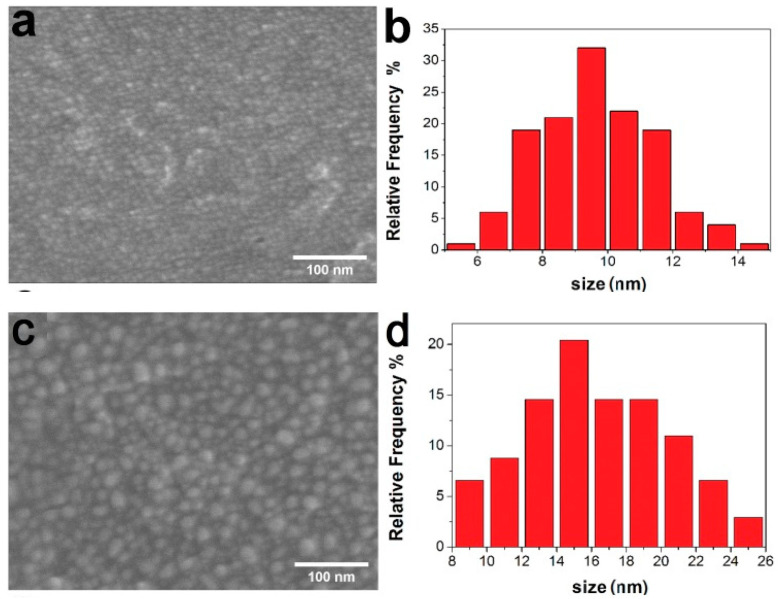
SEM images of Au_CVD2/CoPc (**a**) with the size distribution of AuNPs (**b**) and of Au_CVD3/CoPc (**c**) with the size distribution of AuNPs (**d**).

During the second deposition (the Au_CVD2/CoPc sample, Figure 5a,b), AuNPs coalesced, which was accompanied by a two-fold increase in particle size compared to the Au_CVD1/CoPc sample. The Au content in the Au_CVD2/CoPc sample was 4.9 at% (Figure S1, Supporting Information) in accordance with the EDX spectrum. During the third deposition (the Au_CVD3/CoPc sample, Figure 5c,d), Au nanoclusters consisting of 2–4 particles with sizes from 8 to 26 nm, with predominant sizes of 12–20 nm, were formed on the surface of CoPc films. The Au content in the Au_CVD3/CoPc sample was 7.6 at% (Figure S1, Supporting Information). The average sizes of Au nanoclusters in the heterostructures are summarized in Table 1. Thus, during MOCVD, the growth of AuNPs on CoPc films was accompanied by the nucleation and subsequent fusion of AuNPs with the formation of Au nanoclusters.

### 3.2. Characterization of Heterostructures with AuNPs Obtained by PVD

In the PVD processes, the Au loads were varied from 0.6 to 5 mg in order to obtain samples with concentrations comparable to Au_CVD1/CoPc, Au_CVD2/CoPc, and Au_CVD3/CoPc samples. According to the ICP-AES data, the concentration of gold in Au_PVD1/CoPc was 0.47 ± 0.07 μg/cm^2^ at the Au load of 0.6 mg. The concentrations of gold in Au_PVD2/CoPc and Au_PVD3/CoPc were 0.99 ± 0.09 and 2.1 ± 0.2 μg/cm^2^, respectively (Table 1). As a result, Au_CVD3/CoPc and Au_PVD2/CoPc samples had similar concentrations of gold. 

**Figure 6:**
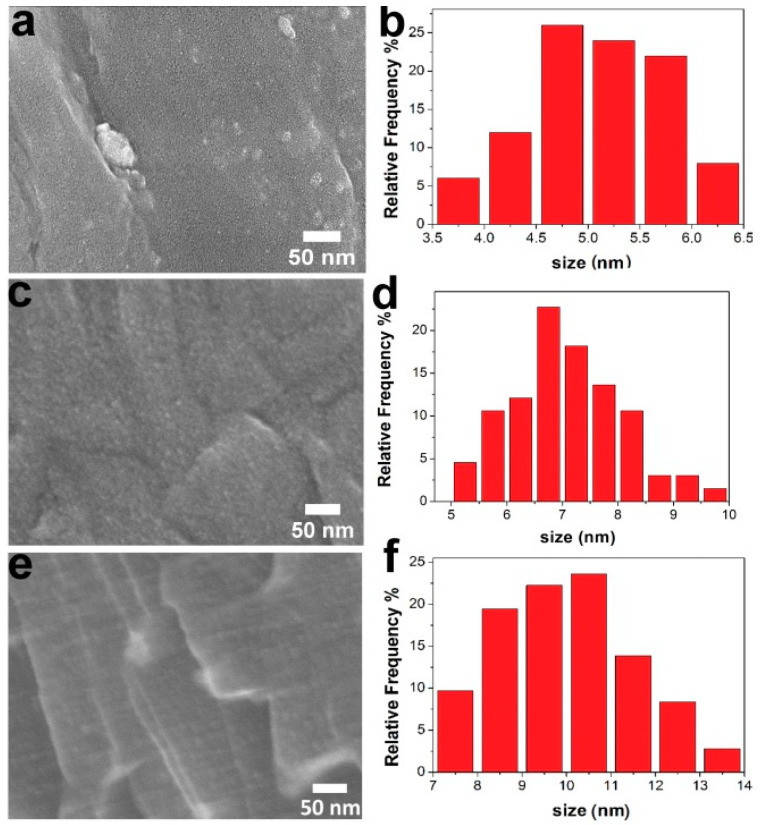
SEM images with the size distribution of AuNPs of Au_PVD1/CoPc (**a**,**b**), Au_PVD2/CoPc (**c**,**d**), and Au_PVD3/CoPc (**e**,**f**).

According to SEM data, ultradispersed AuNPs (sizes up to 6.5 nm) with uniform distribution were formed on the surface of CoPc films after the first deposition (Au_PVD1/CoPc sample, Figure 6a,b). 

After the second deposition (Au_PVD2/CoPc sample, Figure 6c,d), the unimodal distribution of AuNPs on the surface of CoPc film remained, while the average size of AuNPs increased by no more than 1.3–1.4 times compared to that for the Au_PVD1/CoPc sample (Table 1). After the third deposition (sample Au_PVD3/CoPc, Figure 6e,f), Au nanoclusters consisting of 2–3 particles with sizes from 7 to 14 nm, with a predominance of sizes 8–12 nm, formed on the surface of CoPc films. Au content in the Au_PVD1/CoPc, Au_PVD2/CoPc, and Au_PVD3/CoPc samples according to their EDX spectra was 3.7, 6.1 and 9.1 at%, respectively (Figure S2). Thus, the PVD growth of AuNPs on CoPc films was accompanied by the formation of particles with a narrower size distribution compared to the samples obtained by MOCVD.

### 3.3. Characterization of Heterostructures with AuNPs Obtained by DC

**Figure 7:**
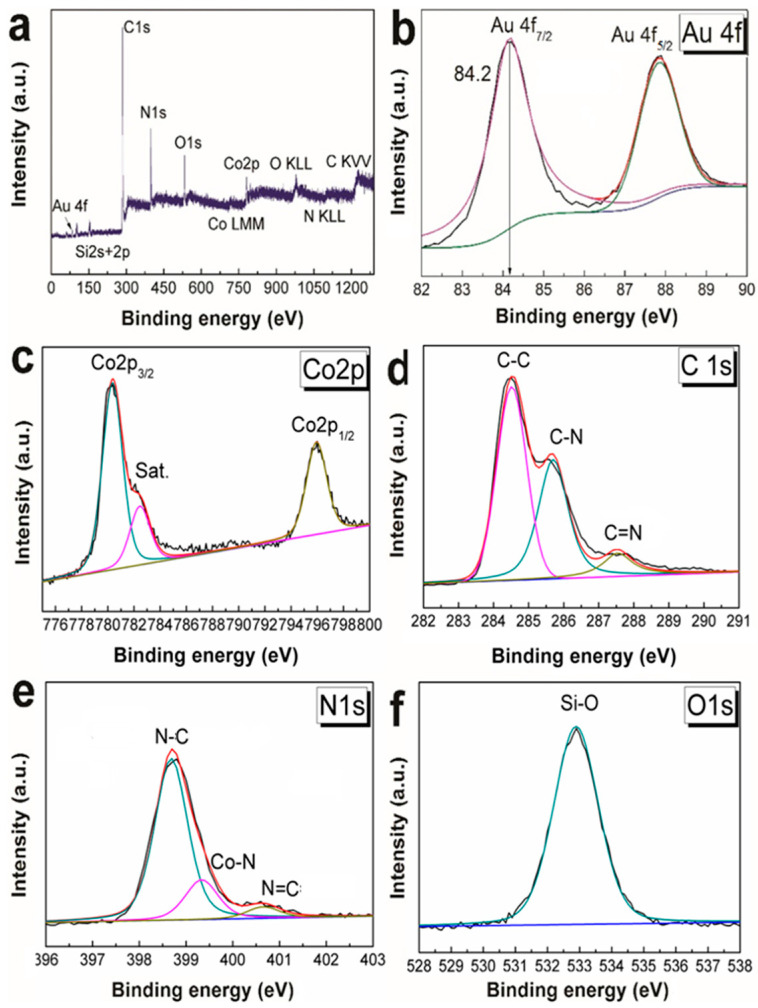
XPS spectra of Au_DC3/CoPc sample (**a**), fitting of Au4f spectra (**b**), fitting of Co2p spectra (**c**), fitting of C1s spectra (**d**), fitting of N1s spectra (**e**), and fitting of O1s spectra (**f**).

During deposition by drop casting, Au concentrations on heterostructures were set experimentally to obtain Au_DC1/CoPc, Au_DC2/CoPc, and Au_DC3/CoPc samples with Au content of 0.33, 0.66, and 0.99 μg/cm^2^, respectively, taking into account the geometric dimensions of the substrates (IDE, working zone S = 0.35 cm^2^) (Table 1). The chemical state of gold in the samples was studied by the XPS method. The XPS spectra of Au_DC3/CoPc are shown in Figure 7 as an example. In the XPS spectrum of Au_DC3/CoPc, along with Au4f lines, intense C1s, N1s, and Co2p lines related to CoPc films, as well as O1s and Si2p lines from the substrate, were observed (Figure 7a).

The positions of Au4f peaks indicate the presence of predominantly metallic gold in Au_DC3/CoPc (Au4f_7/2_ 84.2 eV) with a slight shift in the position of the Au4f_7/2_ peak, which may be associated with the presence of bounded gold (Figure 7b). 

The positions of the Co2p_3/2_ peak (at 780.4 eV), its satellite (at 782.5 eV), and the Co2p_1/2_ peak (at 796.1 eV) correspond to the binding of cobalt with the phthalocyanine ring (Figure 7c). The position of C1s peaks at 284.5 eV (C-C), 285.8 eV (C-N), and 287.5 (C=N) and N1s peaks at 398.3 eV (N-C), 399.5 eV (Co-N), and 400.5 eV (N=C) (Figure 7d,e) confirms the presence of CoPc in the Au_DC3/CoPc sample. In the O1s spectrum (Figure 7f), the peak at 532.9 eV corresponds to Si-O [60].

**Figure 8:**
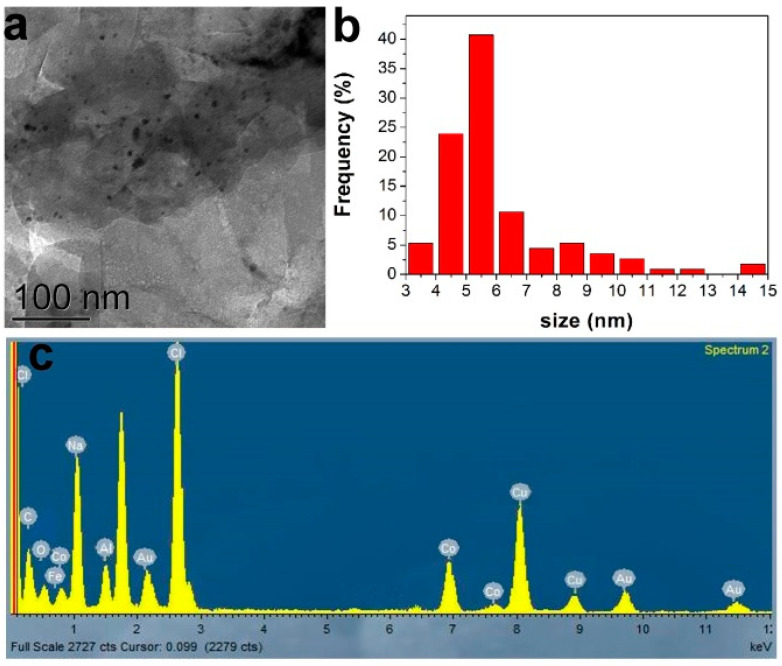
TEM image of Au_DC1/CoPc (**a**) with the size distribution of AuNPs (**b**) and the EDX spectrum of Au_DC1/CoPc (**c**) (Na and Cl refer to the rest of the substrate for the film preparation, Cu lines refer to the TEM grid, and Al lines refer to the crucible for the sample).

Ultradispersed AuNPs are observed on the TEM image of Au_DC1/CoPc (Figure 8a,b). Their sizes (5.5–6 nm) correspond to those in an AuNP solution studied by Eah et al. [56]. In addition to ultradispersed AuNPs, the oval Au nanoclusters with sizes up to 14 nm were also observed on the surface of Au_DC1/CoPc. Unlike Au_CVD1/CoPc and Au_PVD1/CoPc samples, in the case of Au_DC1/CoPc, single AuNPs were scattered on the surface of the CoPc film (Figure 8b). Such a distribution may be due to the diffusion of AuNPs solution into the depth of the CoPc film. The gold content in the Au_CVD1/CoPc sample was 1.9 at.% according to its EDX spectrum (Figure 8c).

**Figure 9:**
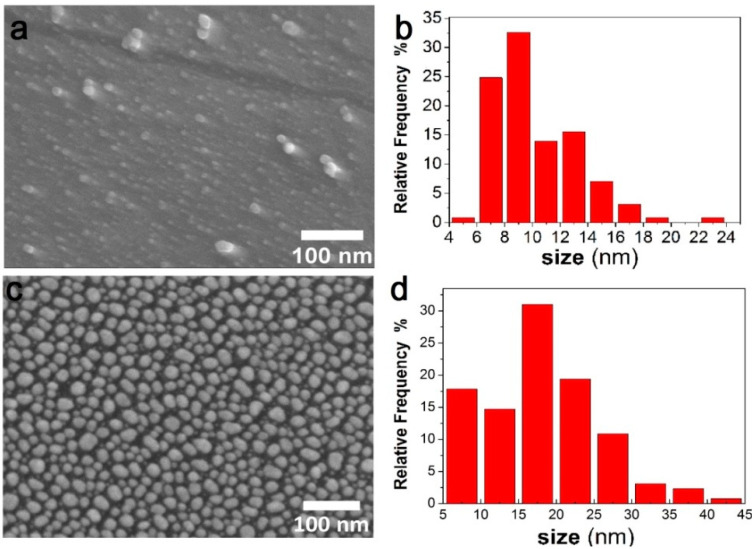
SEM images of Au_DC2/CoPc (**a**) with the size distribution of AuNPs (**b**) and of Au_DC3/CoPc (**c**) with the size distribution of AuNPs (**d**).

After the second DC deposition (sample Au_DC2/CoPc, Figure 9a,b), AuNPs with a bimodal size distribution were observed on the CoPc surface. The size of AuNPs in Au_DC2/CoPc varied from 6 to 24 nm (Table 1), while their distribution became more dense. From the EDX spectrum, the gold content in Au_DC2/CoPc was 3.1 at.% (Figure S3). After the third DC deposition of the AuNPs (sample Au_DC3/CoPc, Figure 9c,d), the surface of the CoPc was covered with Au nanoclusters with a wide size distribution (the predominant sizes were 17–20 nm). The gold content in Au_DC3/CoPc was 5.1 at.% (Figure S3). Thus, the distribution of AuNPs obtained by DC on CoPc films was not homogeneous and was accompanied by their fusion with the formation of Au nanoclusters with sizes up to 45 nm. 

### 3.4. Study of the Sensor Response of AuNP/CoPc Heterostructures to Ammonia and Nitric Oxide

**Figure 10:**
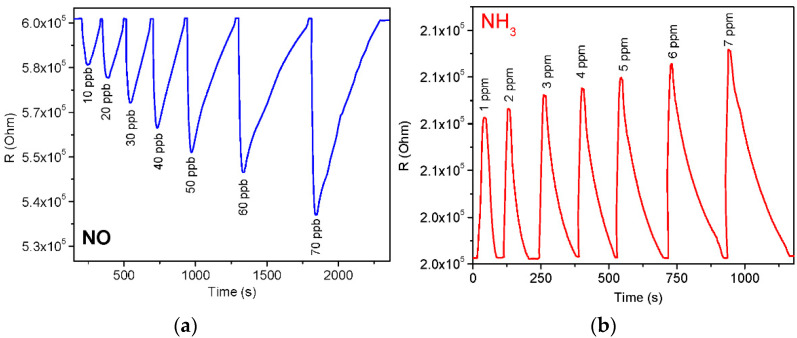
Typical sensor response of an Au_PVD3/CoPc sample to NO (10–70 ppb) (**a**) and NH_3_ (1–7 ppm) (**b**).

The study of the sensor properties of active layers based on CoPc and its heterostructures with AuNPs was carried out by measuring their chemiresistive sensor response to NH_3_ and NO. In this method, the measured value is the resistance (R, Figure 10), which reversibly changes during the interaction of the layer with the gaseous analyte and the subsequent purging. The typical sensor response of an Au_PVD3/CoPc sample to NO (0.1–0.7 ppb) and NH_3_ (1–7 ppm) is presented in Figure 10 as an example. When gaseous NO is introduced into the measuring cell, a sharp decrease in resistance is observed (Figure 10a). The resistance returns to its original value with subsequent purging with argon. In contrast, the introduction of ammonia leads to an increase in the film resistance (Figure 10b). 

This behavior was observed for both bare CoPc films and all investigated heterostructures. Similar behavior was also observed in other works [61,62], where unsubstituted phthalocyanines and their hybrids with carbon nanomaterials, which are p-type semiconductors, were used as active layers. When the adsorption of NH_3_ on the surface occurs, the electron density transfers from the NH_3_ molecule to the CoPc layer. This reduces the concentration of the major charge carriers and increases the resistance. The opposite effect is observed in the case of NO.

To reveal the effect of the deposition of Au nanoparticles on the sensor properties of CoPc films, the sensor response of CoPc films to NH_3_ and NO was compared with that of the heterostructures in which AuNPs were deposited by three different techniques. 

**Figure 11:**
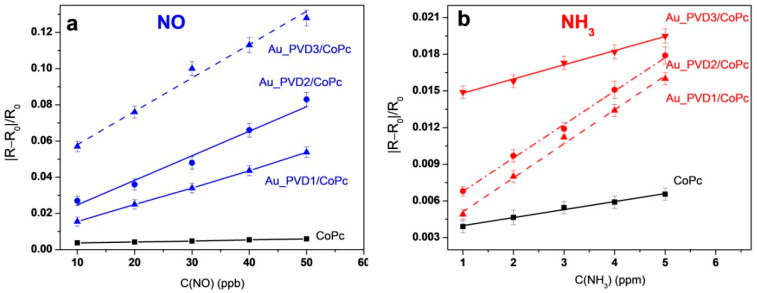
Dependence of the sensor response of CoPc films and heterostructures, in which Au nanoparticles were deposited by the PVD technique, on the concentration of NO (**a**) and NH_3_ (**b**).

Figure 11a,b shows the dependencies of the sensor response of CoPc films and heterostructures, in which Au nanoparticles were deposited by the PVD technique, on the concentration of NO and NH_3_, respectively. It is seen that the deposition of AuNPs on the surface of CoPc films leads to a substantial increase in the sensor response to both gases. For example, the sensor response of Au_PVD1/CoPc to NO (10 ppb) is four times higher compared to a CoPc film. The further increase in the number of nanoparticles in the samples Au_PVD2/CoPc and Au_PVD3/CoPc leads to the 7-fold and 16-fold growth of the sensor response to NO, respectively (Figure 11a). In the case of ammonia detection, the sensor response also increases in the order of CoPc < Au_PVD1/CoPc < Au_PVD2/CoPc < Au_PVD3/CoPc. The value of the response of Au_PVD3/CoPc to NH_3_ (at 2 ppm) is 3.3 times higher than in the case of a CoPc film (Figure 11b). It is important to note that a further increase in the concentration of AuNPs by increasing the number of PVD cycles to four and five does not result in significant growth in the response to both NO and NH_3_. The dependence of the sensor response to NO and NH_3_ on the number of PVD cycles is shown in Figure S4. This means that the number of nanoparticles on the surface of CoPc films has a significant effect on the sensor sensitivity. The Au_PVD3/CoPc heterostructure with the Au content of 2.1 μg/cm^2^ and the average size of Au nanoparticles of 7–14 nm has an optimal composition to ensure the maximum response to both NO and NH_3_. Similar to the data presented by Korotcenkov et al. [63], the growth of the sensor response is observed until a certain concentration of Au nanoparticles is reached. A further increase in the number of nanoparticles can lead to the formation of large clusters and the overgrowth of the film surface, thereby reducing the number of active centers.

**Figure 12:**
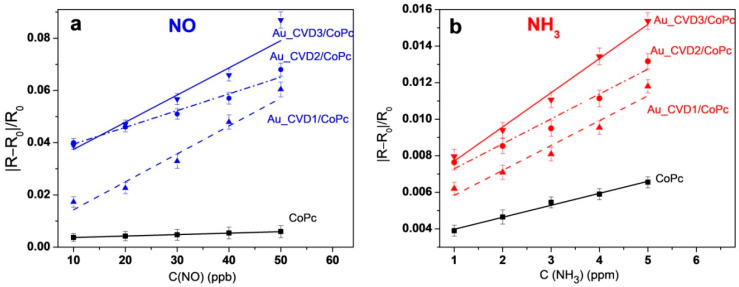
Dependence of the sensor response of CoPc films and heterostructures, in which Au nanoparticles were deposited by the CVD technique, on the concentration of NO (**a**) and NH_3_ (**b**).

Figure 12a,b shows a comparison of the sensor response of CoPc films to NO and NH_3_ with that of heterostructures in which Au nanoparticles were deposited by MOCVD. Similar to heterostructures with AuNPs deposited by PVD, the sensor response of the heterostructure is higher than in the case of CoPc films and increases when the number of nanoparticles grows. The Au_CVD3/CoPc heterostructure demonstrates a 10-fold increase in the sensor response toward NO and a 2-fold increase in the response to NH_3_ compared to the original CoPc film, which is noticeably lower than in the case of similar heterostructures obtained by PVD (see above). Similarly to the case of heterostructures prepared by PVD, the further increase in the number of CVD cycles does not lead to an increase in the sensor response (Figure S5).

**Figure 13:**
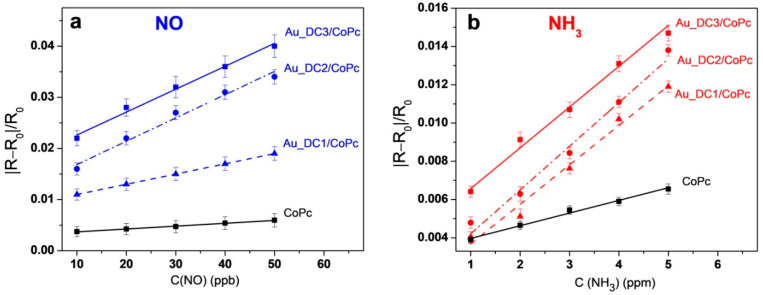
Dependence of the sensor response of CoPc films and heterostructures, in which Au nanoparticles were deposited by the drop-casting technique, on the concentration of NO (**a**) and NH_3_ (**b**).

The sensor response of heterostructures in which different concentrations of AuNPs were deposited by drop casting was also compared. Figure 13a,b shows the same trend as in the case of heterostructures prepared by PVD and CVD methods: the sensor responses toward both NO and NH_3_ increase in the order of CoPc < Au_DC1/CoPc < Au_DC2/CoPc < Au_DC3/CoPc. In the case of NO (10 ppb) detection, the response of Au_DC3/CoPc is about six times higher than that of the CoPc film, while the response toward NH_3_ (2 ppm) shows an increase of only two times. 

### 3.5. Comparison of the Sensor Properties of Heterostructures with AuNPs Deposited in Different Ways 

**Figure 14:**
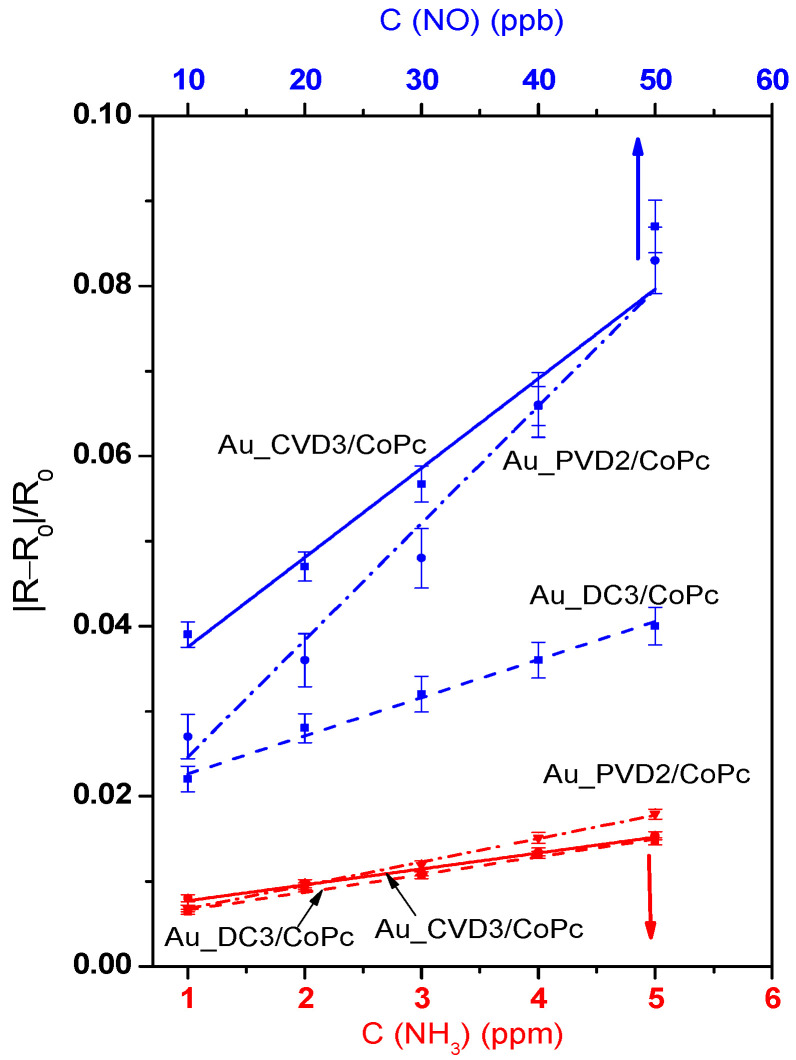
Dependence of the sensor response of Au_PVD2/CoPc, Au_CVD3/CoPc, and Au_DC3/CoPc on NO (blue lines) and NH_3_ (red lines) concentrations.

The comparative analysis described above showed that in the case of heterostructures in which nanoparticles were deposited in three different ways, a similar trend is observed, namely, when the concentration of nanoparticles increases to a certain value, the sensor response to both ammonia and nitric oxide increases. To understand which method is the most preferable in terms of increasing the sensitivity of the active layer, the sensor characteristics of heterostructures containing a similar amount of AuNPs were compared. As shown above, the heterostructures Au_PVD2/CoPc, Au_CVD3/CoPc, and Au_DC3/CoPc have almost the same concentration of gold. The dependencies of the sensor response for Au_PVD2/CoPc, Au_CVD3/CoPc, and Au_DC3/CoPc samples on the concentrations of NO and NH_3_ are shown in Figure 14. Figure 14 shows that Au_CVD3/CoPc demonstrates the highest sensitivity to NO in the concentration range from 10 to 30 ppb. At 40–50 ppm NO, the difference between the sensor responses of Au_CVD3/CoPc and Au_PVD2/CoPc is within the measurement error. Thus, Au_PVD2/CoPc and Au_CVD3/CoPc samples, which are characterized by a narrow distribution of small sizes of AuNPs or Au nanoclusters, have the highest sensor responses to NO. The heterostructure Au-PVD2/CoPc with a particle size of 8 nm is characterized by the most rapid increase in response with an increase in NO concentration. According to [63], the smaller the Au nanoparticles, the greater their activity in charge transfer processes, as well as in the adsorption and desorption reactions of molecular gases.

In the case of nanoparticles obtained by the DC method, the sensor response to nitric oxide is 2–3 times lower compared to the heterostructures obtained by the other two methods. This may be due to the possible presence of an organic shell of AuNPs necessary for their stabilization when they are obtained by the wet method. A similar trend is also observed when comparing the sensor response of heterostructures with gold nanoparticles of the same size.

Interestingly, unlike NO, the sensor response to NH_3_ is practically the same for heterostructures in which nanoparticles were obtained by vapor deposition and wet methods. As has already been mentioned above, these heterostructures had almost the same concentration of gold but different sizes and distribution of AuNPs (Table 1). This behavior may be related to the different mechanisms of interaction of NO and NH_3_ with heterostructures based on phthalocyanine and gold nanoparticles, which are discussed below.

The improvement in the sensitivity of the Au/CoPc heterostructures to both gases with an increase in the concentration of AuNPs may be associated with the sensitization mechanism. Based on the literature analysis [63,64,65,66,67], we believe that in Au/CoPc heterostructures the charge transfers from CoPc to Au and makes AuNPs negatively charged, increasing the number of hole carriers on the CoPc surface. Note that the concentration of hole carriers decreases with the adsorption of donor gas, but increases with the adsorption of acceptor gas [65,68]. Comparing the two sensitization mechanisms described in the literature [65], we can assume that in the case of Au/CoPc heterostructures, the most likely mechanism is electronic sensitization, in which the main role of Au is to increase the number of hole carriers. The mechanism of chemical sensitization based on the formation of O_2_^−^(_ad._) species that would react with NH_3_(_ad._), giving electrons back to CoPc, seems unlikely here due to the low operating temperatures of the sensor (50 °C).

The difference in the behavior of NO and NH_3_ gases may be related to the stronger binding of NO with AuNPs. According to the work of Chinh et al. [69], in Au/ZnO heterostructures, NO molecules can adsorb on Au cores, forming NO^−^_(ad.)_ species, according to Equation (1):

NO_(g)_ + e = NO^−^_(ad.)_ (1)

In this case, the sensor response is the result of a dynamic equilibrium between NO adsorption and NO^−^_(ad)_ desorption. Thus, the role of Au nanoparticles is to reduce the energy barrier of adsorption and desorption for NO and generate NO^−^_(ad.)_ species. 

Recently, Korotcenkov et al. [63,64] described the mechanism of chemical sensitization of gold-containing heterostructures based on the generation of O^−^_(ad.)_ species near Au clusters, which then further react with gases. Note that the mechanism of chemical sensitization depends entirely on the efficiency of generation and the availability of these species [63,65,66,70]. Apparently, in the case of the studied heterostructures, mixed sensitization occurs, which is based on the generation of NO^−^_(ad.)_ species that can migrate to hole carriers in the CoPc film. In this case, the adsorption/desorption processes that determine gas sensitivity have an activation character, and the parameters describing these processes depend on the grain size and distribution [66]. Thus, the sensor response of Au/CoPc heterostructures to NO depends not only on the concentration of gold but also on the size of the AuNPs and distribution parameters, which are responsible for the efficiency of the generation and migration of NO^−^_(ad.)_ species.

### 3.6. Investigation of the Sensor Characteristics of AuNP/CoPc Heterostructures

**Figure 15:**
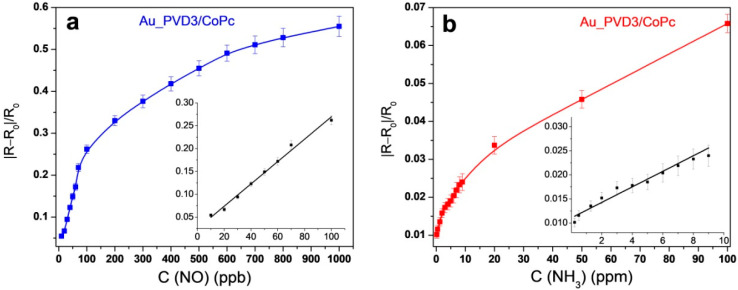
Dependences of the sensor response of Au_PVD3/CoPc on the concentration of NO (10–1000 ppb) (**a**) and NH_3_ (0.5–100 ppm) (**b**).

Au_PVD3/CoPc was shown above to exhibit the best sensor response to both ammonia and nitric oxide among the investigated heterostructures. For this reason, it were chosen for a more detailed investigation of its sensor characteristics and to assess the possibility of its use for detecting low concentrations of ammonia and nitric oxide in exhaled air. The sensitivity of the Au_PVD3/CoPc sensor to NO and NH_3_ was tested in a wider range of concentrations. The dependences of the sensor response on the concentration of NO (10–1000 ppb) and NH_3_ (0.5–100 ppm) are shown in Figure 15a,b. 

The dependence of the response on the concentration of NO was linear in the range from 10 to 100 ppb, while on the concentration of NH_3_ the dependence was linear from 0.5 to 10 ppm. Both of these intervals cover the concentration range important for determining these gases in the exhaled air [10]. The limit of detection (LOD) of NO, defined as *3σ/m*, where *σ* is the standard deviation of the sensor response to 10 ppm NH_3_ and *m* is the slope of the corresponding calibration plot (Figure 15) in the linear region, was 4 ppb, while the response and recovery times of the sensor, determined at 10 ppb of NO, were 40 s and 100 s, respectively. The LOD of NH_3_ was 0.1 ppm; the response and recovery times determined at 1 ppm of NH_3_ were 20 and 85 s, respectively.

**Figure 16:**
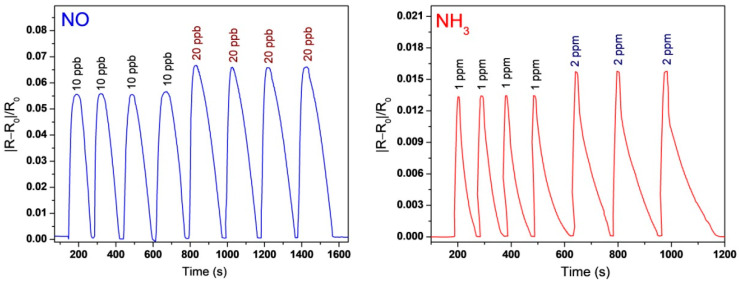
Repeatability of the Au_PVD3/CoPc sensor.

The reproducibility of the response was studied by repeated exposure to different concentrations of the investigated analytes, as shown in Figure 16. Au_PVD3/CoPc sensing layers showed very good repeatability of the sensor response to both ammonia and nitric oxide. Because the operating temperature is a unique characteristic of each sensor, the sensor response of a Au_PVD3/CoPc layer toward NH_3_ and NO was tested at temperatures from 40 to 100 °C (Figure S6). An increase in the temperature to 70 °C practically does not affect the sensor response to both gases, while its further growth to 100 °C leads to an increase in the response. This behavior appears to be due to the growth of the concentration of charge carriers in p-type semiconductors with temperature. 

**Figure 17:**
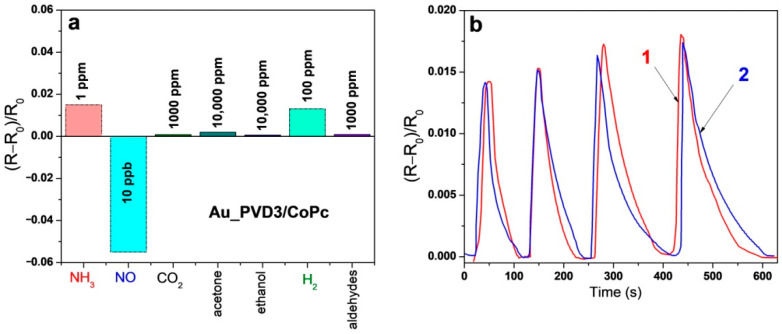
(**a**) Diagram of selectivity of Au_PVD3/CoPc sensing layers toward various analytes. (**b**) Sensor response of Au_PVD3/CoPc to ammonia (1–4 ppm) in air (1) and in a mixture of gases with a composition close to exhaled air (N_2_—74%, O_2_—16%, H_2_O—6%, and CO_2_—4%) (2).

Selectivity is another parameter important for the practical application of chemical sensors. To investigate the selectivity, the response of the Au_PVD3/CoPc layers to various gaseous analytes was tested (Figure 17a). It is obvious that the response of sensing layers to both NH_3_ and NO gases was much higher than to interfering analytes such as carbon dioxide, acetone, ethanol, hydrogen, and a mixture of aldehydes (butanal, heptanal, and octanal). It is necessary to mention that the concentration of ammonia and nitric oxide given in the diagram is much less than that of the other volatile organic analytes. These results make the investigated sensing layers attractive for detecting ammonia and nitric oxide in gas mixtures. 

To demonstrate the possible application of the investigated heterostructures for the detection of ammonia in exhaled air, the sensor response of Au_PVD3/CoPc was tested in a mixture of gases with a composition close to the exhaled air of healthy people (N_2_—74%, O_2_—16%, H_2_O—6%, and CO_2_—4%). For this purpose, small amounts of ammonia (1–4 v.%) were added to the preliminarily prepared gas mixture (N_2_—74%, O_2_—16%, H_2_O—6%, and CO_2_—4%). The sensor response of Au_PVD3/CoPc to ammonia (1–4 ppm) in the gas mixture was compared with that to ammonia in air, as shown in Figure 17b. It was shown that the determination of ammonia in a mixture similar in composition to exhaled air did not lead to a significant change (within the measurement error) in the sensor response to ammonia measured in air. Thus, it was shown that the investigated heterostructures are promising materials for use as the active layers of sensors for determining low concentrations of ammonia in exhaled air. 

Due to the low stability of NO in air, its determination in exhaled air samples is possible only with strict control of the inert atmosphere. For this reason, we did not determine gaseous NO in the gas mixture close to exhaled air. It has been shown that Au/CoPc hybrid materials are very promising for the determination of nitric oxide. The properties of the sensors studied in this work are superior in some characteristics to the sensors described in recent years (Table 2). They have high sensitivity to NO, good selectivity, relatively short response and recovery times, and, importantly, they operate at significantly lower temperatures with a low detection limit. To detect NO in oxygen- and water-containing gas mixtures, some researchers first quantitatively oxidized NO to NO_2_, and then conducted a study of the sensor response to NO_2_. The use of this approach will be the subject of a separate publication.

## 4. Conclusions

In this work, new heterostructures based on cobalt phthalocyanine (CoPc) films decorated with gold nanoparticles (AuNPs) were obtained. The AuNPs were deposited on the surface of CoPc films by gas-phase methods (MOCVD, PVD) and drop casting (DC). Within each method, AuNPs were deposited onto CoPc films one, two, or three times sequentially to provide a proportional increase in the concentration of gold in Au/CoPc heterostructures. Au/CoPc heterostructures with a narrow distribution of small sizes of AuNPs were obtained by gas-phase methods, while the drop casting resulted in the production of Au/CoPc heterostructures with a scattered or inhomogeneous distribution of AuNPs. 

The effect of gold concentration and the size of AuNPs on the sensor response of Au/CoPc heterostructures to NH_3_ (1–10 ppm) and NO (10–50 ppb) was investigated. Regardless of the fabrication method, the response of Au/CoPc heterostructures to NH_3_ and NO gases increased with an increase in the concentration of gold. The sensor response of Au/CoPc heterostructures to NH_3_ increased 2–3.3 times compared to CoPc film, whereas in the case of NO it increased up to 16 times. A comparison of Au/CoPc samples with the same concentration of gold (~1 μg/cm^2^) deposited by different methods revealed the influence of AuNPs morphology on the sensor responses of these heterostructures to NO. In particular, the Au/CoPc structure with the smallest AuNPs size of 8 nm, obtained by the PVD method, was characterized by the most rapid increase in the sensor response with an increase in NO concentration. The detection limits of the Au/CoPc heterostructure with the highest gold content (~2.1 µg/cm^2^) to NH_3_ and NO were 0.1 ppm and 4 ppb, respectively. The recovery times determined at 1 ppm NH_3_ and 10 ppb NO were 85 and 100 s, respectively. It was shown that Au/CoPc heterostructures can be used for the detection of NH_3_ in a gas mixture simulating exhaled air (N_2_—74%, O_2_—16%, H_2_O—6%, and CO_2_—4%). This makes the studied heterostructures promising materials for further investigation as active layers of sensors or sensor arrays for determining low concentrations of ammonia in exhaled air, which is a biomarker of some kidney diseases. Further research in this area should focus on improving the selectivity of layers and the creation of miniature sensor devices based on the studied heterostructures for the determination of NH_3_ and NO in real exhaled air samples.

## Figures and Tables

**Table 1:** Au content and average sizes of Au nanostructures in the Au/CoPc heterostructures.

Series	Sample Designation	Au Content, μg/cm^2^	Average Sizes of Au Nanostructures, nm
Au_MOCVD	Au_CVD1/CoPc	0.34 ± 0.05	1–7
	Au_CVD2/CoPc	0.64 ± 0.08	6–13
	Au_CVD3/CoPc	1.1 ± 0.1	8–26
Au_PVD	Au_PVD1/CoPc	0.47 ± 0.07	3–7
	Au_PVD2/CoPc	0.99 ± 0.09	5–10
	Au_PVD3/CoPc	2.1 ± 0.2	7–14
Au_DC	Au_DC1/CoPc	0.33 ± 0.05	3–14
	Au_DC2/CoPc	0.66 ± 0.05	4–24
	Au_DC3/CoPc	0.99 ± 0.09	5–45

**Table 2:** Main characteristics of chemiresistive sensors based on metal oxides, phthalocyanines, and nanoparticles toward NO.

Sensing Material	Operating Temperature, °C	NO Concentration Range, ppm	Sensitivity (S = R/R_0_)	Recovery Time, min	Ref.
NiPc	160	5–500	41.3–42.3 (5–50 ppm)	20	[49]
Ag-doped WO_3_-based powder sensor	300	40–100	21.5 (40 ppm)	˃10 (40 ppm)	[71]
WO_3_/Au layer	200	0–440	78 (440 ppm)	4.1 (440 ppm)	[72]
Iron oxide nanorods	250	0.5–2.75	15.2 (2.75 ppm)	<1 (2.75 ppm)	[73]
ZnO film	150	0.07–0.1	16.9 (100 ppb)	8.3 (100 ppb)	[74]
Au-PVD3/CoPc	50	0.01–0.1	26.2 (100 ppb)	9 (100 ppb)	This work

## Data Availability

Not applicable.

## Supplementary Materials

The following supporting information can be downloaded at: https://www.mdpi.com/article/10.3390/bios12070476/s1, Figure S1. EDX spectra of Au-CVD2/CoPc (a) and Au-CVD3/CoPc (b); Figure S2. EDX spectra of Au-PVD1/CoPc (a), Au-PVD2/CoPc (b), and Au-PVD3/CoPc (c). Figure S3. EDX spectra of Au-DC2/CoPc (a) and Au-DC3/CoPc (b). Figure S4. Dependence of the sensor response of heterostructures, in which Au nanoparticles were deposited by the PVD technique, on the number of PVD cycles. Figure S5. Dependence of the sensor response of heterostructures, in which Au nanoparticles were deposited by the CVD technique, on the number of CVD cycles. Figure S6. Dependence of the response of Au_PVD3/CoPc sensor on temperature.
