# Supplementary material for: Heterostructures Based on Cobalt Phthalocyanine Films Decorated with Gold Nanoparticles for the Detection of Low Concentrations of Ammonia and Nitric Oxide
Source: Biosensors (Basel). 2022 Jun 30;12(7):476. doi: 10.3390/bios12070476 (PMC9313448; doi:10.3390/bios12070476)
Supplement: Supplementary file 1 [file biosensors-12-00476-s001.zip › biosensors-1765610-supplementary.pdf]

Supporting Information

Heterostructures Based on Cobalt Phthalocyanine Films Decorated with Gold Nanoparticles for the Detection of Low Concentrations of Ammonia and Nitric Oxide

Svetlana I. Dorovskikh [1], Darya D. Klyamer [1], Eugeny A. Maksimovskiy [1], Victoria V. Volchek [1], Sergey M. Zharkov [2,3], Natalia B. Morozova [1] and Tamara V. Basova [1,*]

[1] Nikolaev Institute of Inorganic Chemistry SB RAS, 3 Lavrentiev Pr., Novosibirsk 630090, Russia; reter16@yandex.ru (S.I.D.); klyamer@niic.nsc.ru (D.D.K.); eugene@niic.nsc.ru (E.A.M.); volchek@niic.nsc.ru (V.V.V.); mor@niic.nsc.ru (N.B.M.)

[2] Kirensky Institute of Physics, Federal Research Center KSC SB RAS, Krasnoyarsk 660036, Russia; zharkov@iph.krasn.ru

[3] Siberian Federal University, Laboratory of electron microscopy, Krasnoyarsk 660041, Russia

* Correspondence: basova@niic.nsc.ru

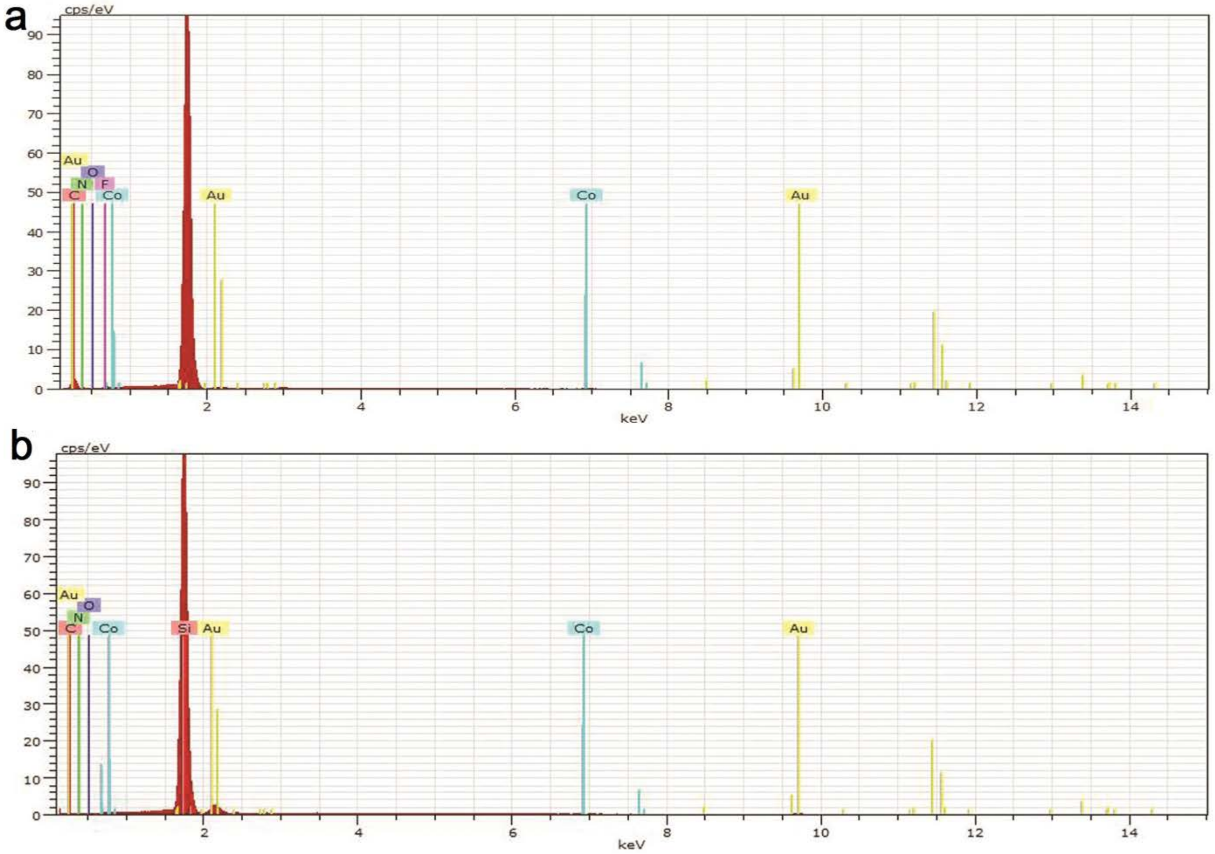

Figure S1. EDX spectra of Au_CVD2/CoPc (a) and Au_CVD3/CoPc (b).

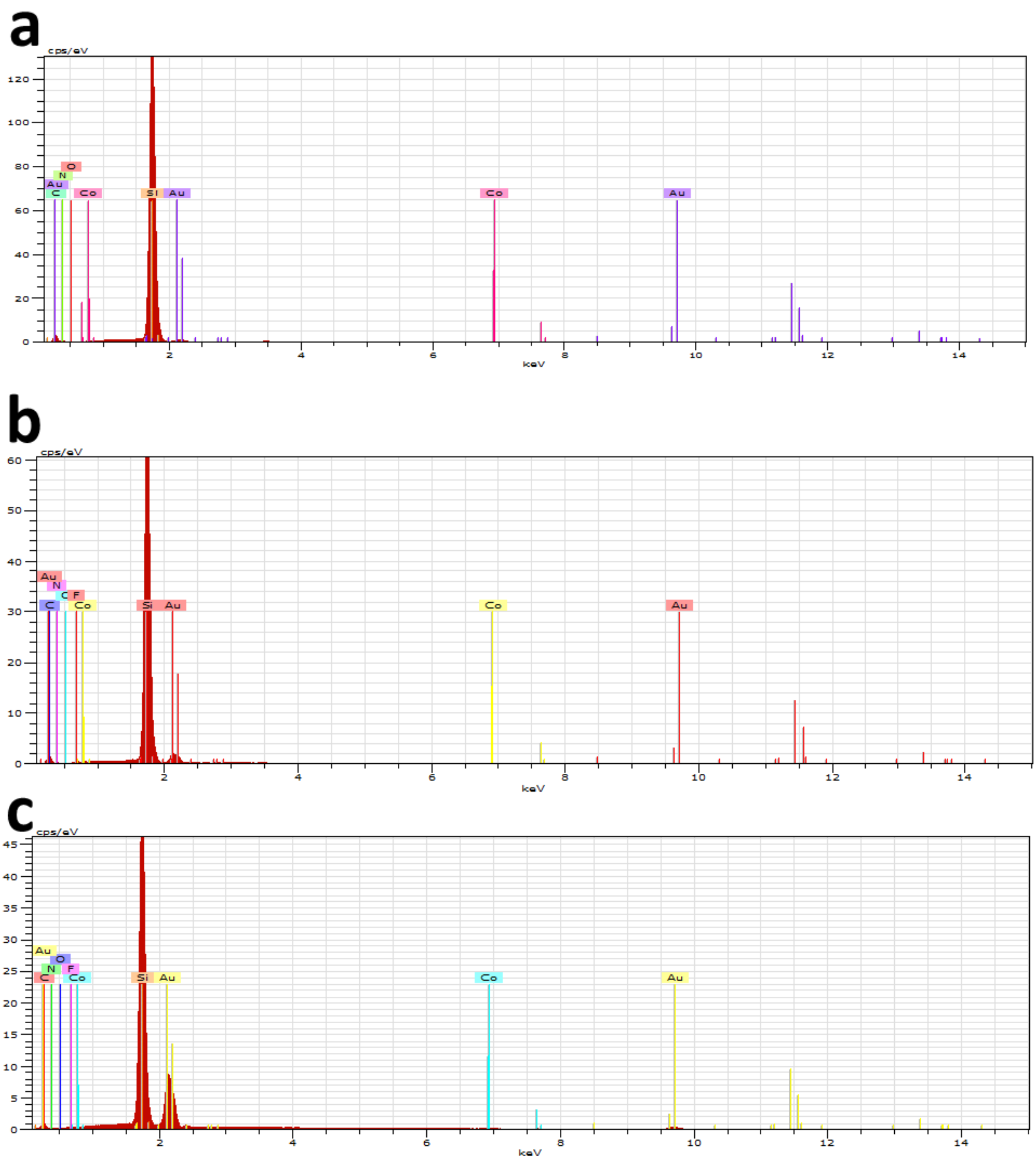

Figure S2. EDX spectra of Au_PVD1/CoPc (a), Au_PVD2/CoPc (b), and Au_PVD3/CoPc (c).

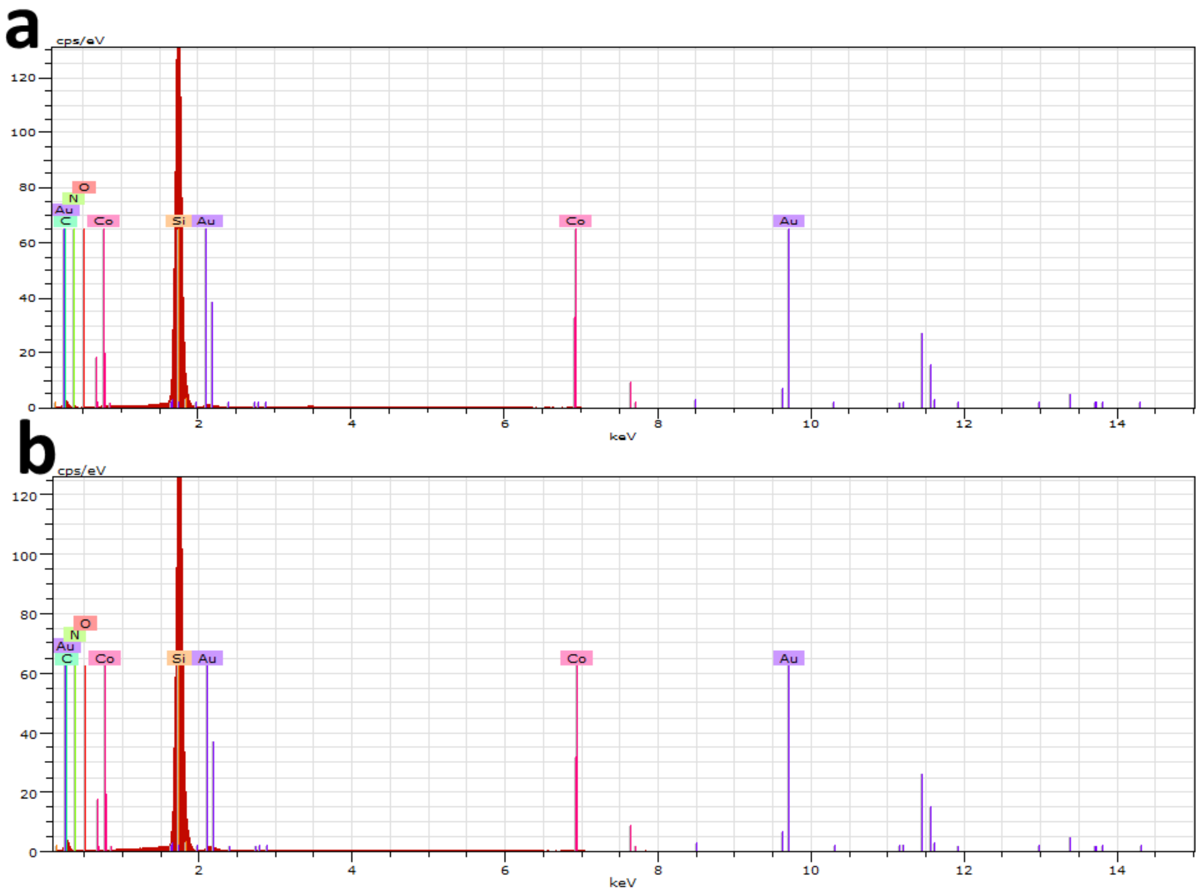

Figure S3. EDX spectra of Au_DC2/CoPc (a) and Au_DC3/CoPc (b).

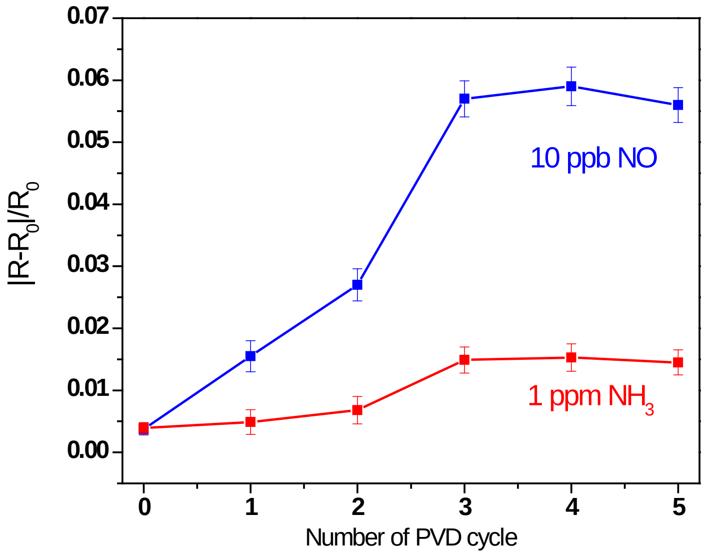

Figure S4. Dependence of the sensor response of heterostructures, in which Au nanoparticles were deposited by a PVD technique, on the number of PVD cycles.

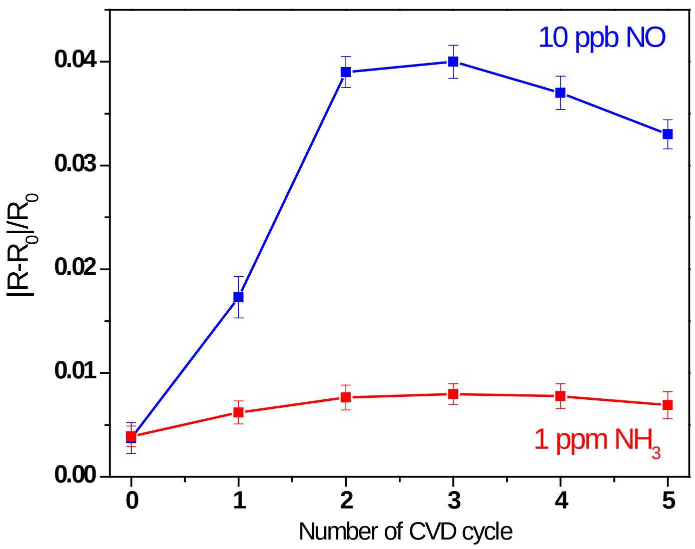

Figure S5. Dependence of the sensor response of heterostructures, in which Au nanoparticles were deposited by a CVD technique, on the number of CVD cycles.

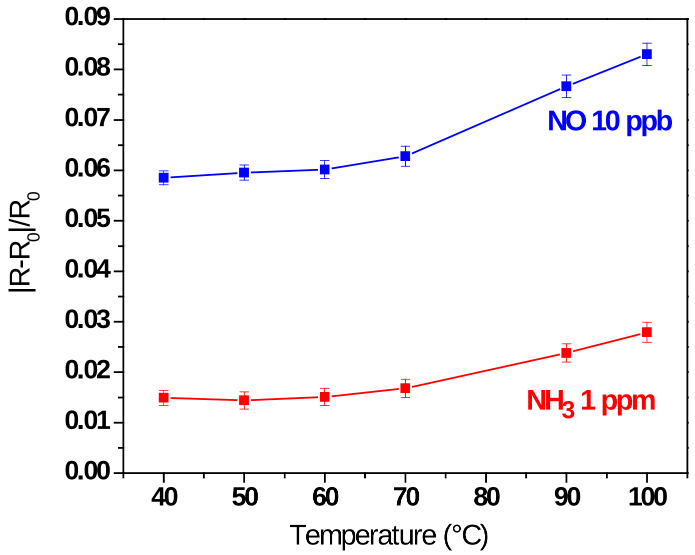

Figure S6. Dependence of the response of Au_PVD3/CoPc sensor on temperature.
